# TGF-β in the Secretome of Irradiated Peripheral Blood Mononuclear Cells Supports In Vitro Osteoclastogenesis

**DOI:** 10.3390/ijms21228569

**Published:** 2020-11-13

**Authors:** Layla Panahipour, Zahra Kargarpour, Maria Laggner, Michael Mildner, Hendrik J. Ankersmit, Reinhard Gruber

**Affiliations:** 1 Department of Oral Biology, University Clinics for Dentistry, Medical University of Vienna, Sensengasse 2a, 1090 Vienna, Austria; layla.panahipour@meduniwien.ac.at (L.P.); zahra.kargarpooresfahani@meduniwien.ac.at (Z.K.); 2 Laboratory for Cardiac and Thoracic Diagnosis, Regeneration and Applied Immunology, Währingergürtel 18-20, 1090 Vienna, Austria; maria.laggner@meduniwien.ac.at (M.L.); hendrik.ankersmit@meduniwien.ac.at (H.J.A.); 3 Division of Thoracic Surgery, Medical University of Vienna, Währingergürtel 18-20, 1090 Vienna, Austria; 4 Research Division of Biology and Pathobiology of the Skin, Department of Dermatology, Medical University of Vienna, Währingergürtel 18-20, 1090 Vienna, Austria; michael.mildner@meduniwien.ac.at; 5 Department of Periodontology, School of Dental Medicine, University of Bern, Freiburgstrasse 7, 3010 Bern, Switzerland; 6 Austrian Cluster for Tissue Regeneration, Donaueschingenstraße 13, 1200 Vienna, Austria

**Keywords:** wound healing, secretome, osteoclastogenesis, macrophages, apoptosis, necroptosis, periodontitis

## Abstract

Osteoclastogenesis required for bone remodeling is also a key pathologic mechanism of inflammatory osteolysis being controlled by paracrine factors released from dying cells. The secretome of irradiated, dying peripheral blood mononuclear cells (PBMCs) has a major impact on the differentiation of myeloid cells into dendritic cells, and macrophage polarization. The impact on osteoclastogenesis, however, has not been reported. For this aim, we used murine bone marrow macrophages exposed to RANKL and M-CSF to initiate osteoclastogenesis, with and without the secretome obtained from γ-irradiated PBMCs. We reported that the secretome significantly enhanced in vitro osteoclastogenesis as determined by means of histochemical staining of the tartrate-resistant acid phosphatase (TRAP), as well as the expression of the respective target genes, including TRAP and cathepsin K. Considering that TGF-β enhanced osteoclastogenesis, we confirmed the TGF-β activity in the secretome with a bioassay that was based on the increased expression of IL11 in fibroblasts. Neutralizing TGF-β by an antibody decreased the ability of the secretome to support osteoclastogenesis. These findings suggested that TGF-β released by irradiated PBMCs could enhance the process of osteoclastogenesis in vitro.

## 1. Introduction

Osteoclastogenesis, the formation of bone-resorbing cells of the hematopoietic lineage, is a central mechanism for removing damaged bone to be replaced by new bone by osteoblasts [1]. Osteoclastogenesis becomes a pathological process when bone resorption exceeds bone formation, which is the hallmark of systemic metabolic bone diseases such as osteoporosis [2] and the local inflammatory osteolysis, as observed in periodontitis and periimplantitis [3,4]. Imbalanced bone remodeling, as well as inflammatory osteolysis, are both controlled by paracrine signals, including those originating from dying cells. It is particularly the apoptotic [5,6], senescent [7], and necrotic osteocytes [6] that signal the need to resorb the damaged bone areas that might be infected by oral pathogens, for instance, in periodontitis. This mechanism of controlling local osteoclastogenesis is not restricted to osteocytes as also senescent fibroblasts can drive the process of osteoclastogenesis [8]. There might even be a central molecular mechanism linking cells dying because of chronic inflammation, radiation therapy or aging, and the formation of osteoclasts. One central principle to sustain homeostasis is that the dying cells release metabolites as ‘goodbye’ signals that help to maintain tissue integrity [9].

Transforming growth factor beta-1 (TGF-β1) is possibly among these ‘goodbye’ signals that are released by dying cells, being a potent enhancer of RANKL (Receptor activator of nuclear factor kappa-Β ligand, also known as tumor necrosis factor ligand superfamily member 11)-induced osteoclastogenesis in bone marrow cultures [10]. TGF-β is active in the supernatant of apoptotic tumor-associated macrophages [11] and released by apoptotic T cells [12]. Additionally, leucocyte apoptosis upon the storage of whole blood facilitates the release of TGF-β [13]. Thus, it is likely that dying cells release TGF-β that, in turn, supports the process of osteoclastogenesis, at least in vitro. With respect to oral biology, dying cells are found in the lamina propria, and to a lesser extent, in the sulcular epithelium of human periodontitis lesions [14], and in rodent periodontitis models [15,16,17]. Moreover, apoptotic macrophages are present in periodontitis and gingivitis tissue [14]. Obviously, this scenario is not restricted to periodontitis lesions. Thus, there is reason to ask if TGF-β being among the paracrine signals released by dying leucocytes is capable of supporting the process of osteoclastogenesis.

To test this assumption, we can take advantage of the secretome of irradiated peripheral blood mononuclear cells (PBMCs) that underwent apoptotic and necroptotic cell death [18]. The secretome of irradiated PBMCs contains a large spectrum of paracrine signals that hold a positive function in vivo: in myocarditis [19], chronic heart failure [20], spinal cord injury [21], stroke [22], and wound healing [23]. Processing of the secretome has advanced towards medicinal products, including viral clearance [24] and good manufacturing practice [25]. Phase I clinical trial on the secretome confirmed pre-clinical findings [26] when no cutaneous adverse events were observed in healthy volunteers (ClinicalTrials.gov; Identifier: NCT02284360) [27]. Today, a phase II clinical trial focused on the secretome to promote wound closure of diabetic foot ulcers (EudraCT number 2018-001,653-27). However, it required in vitro studies to uncover the underlying cellular mechanisms, and more generally, to learn how the paracrine environment of dying PBMCs affects basic cellular mechanisms such as osteoclastogenesis. 

In vitro, the secretome of irradiated PBMCs suppressed the development of dendritic cells [28] and the anti-inflammatory activity on macrophages, indicated by an M1-to-M2 shift [29]. It was particularly the lipid fraction of the secretome that was identified to mediate the indicated effects [28,29]. Dendritic cells and macrophages both originate from hematopoietic cells, similar to the osteoclasts [30]. Thus, and considering that blocking of dendritic cells formation and the suppression of M1 macrophages can open the door for the differentiation of the hematopoietic progenitors towards the osteoclastogenic lineage, it can be assumed that the secretome of irradiated PBMCs also affects the formation of osteoclasts, possibly involving its TGF-β activity [27,31]. To test this assumption, we performed bone marrow culture where hematopoietic cells were in the presence of RANKL and macrophage colony-stimulating factor (M-CSF) [32]. Under these conditions, cells differentiate into multinucleated cells staining positive for the tartrate-resistant acid phosphatase (TRAP), also expressing the marker genes TRAP and cathepsin K (CTSK) [33]. In support of our assumption, we showed that the secretome of irradiated PBMCs indeed enhanced the process of osteoclastogenesis involving TGF-β activity.

## 2. Results

### 2.1. Secretome Increased the Formation of TRAP^+^ Multinucleated Cells in Bone Marrow Cultures

**Figure 1:**
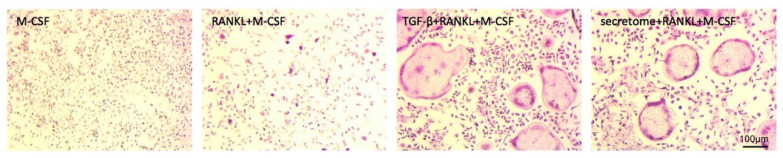
The secretome increased the formation of TRAP^+^ multinucleated cells in bone marrow cultures. Murine bone marrow-derived macrophages were exposed to the secretome corresponding to 1 × 10^6^ irradiated PBMCs/mL in the presence of 30 ng/mL RANKL and 20 ng/mL M-CSF for five days. The histochemical staining identified the cells staining positive for the tartrate-resistant acid phosphatase (TRAP^+^). The multinucleated cells with more than three nuclei and red stain were considered “osteoclast-like cells,” even though mononuclear cells also showed positive TRAP staining in the presence of RANKL and particularly when combined with 10 ng/mL TGF-β or the secretome.

The secretome of PBMCs previously showed to suppress the differentiation of hematopoietic cells towards the dendritic lineage [28] and to shift macrophages from M1-to-M2 [29], together suggesting a potent impact on the lineage decision of hematopoietic cells. Considering that osteoclasts also belong to the hematopoietic lineage, it might be feasible that the secretome of PBMCs affects osteoclastogenesis. To test this assumption, we grew primary macrophages in the presence of M-CSF and RANKL to initiate osteoclastogenesis, with and without the secretome of γ-irradiated PBMCs or recombinant TGF-β1. We reported that the secretome of γ-irradiated PBMCs corresponding to 1 × 10^6^ cells/mL, similar to TGF-β1, significantly increased the formation of TRAP^+^ multinucleated cells, suggesting an enhanced in vitro osteoclastogenesis (Figure 1). 

### 2.2. Secretome Increased the Expression of Osteoclast Marker Genes in Bone Marrow Cultures

**Figure 2:**
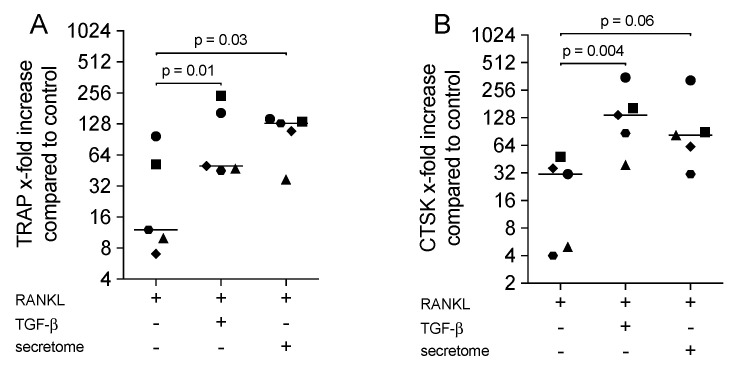
Secretome increased the expression of osteoclast marker genes in bone marrow cultures. Murine bone marrow-derived macrophages were exposed to the secretome corresponding to 1 × 10^6^ irradiated PBMCs/mL or 10 ng/mL TGF-β1 in the presence of 30 ng/mL RANKL and 20 ng/mL M-CSF for five days. X-fold change of TRAP (**A**) and CTSK (**B**) expression compared to M-CSF control was indicated. Dot plots represented independent experiments (*n* = 5). *p*-values were calculated by the Friedman test.

To further confirm that the secretome of γ-irradiated PBMCs supports osteoclastogenesis, the expression of genes that exert an essential function in osteoclasts was determined. Consistent with the histochemical staining, the secretome and recombinant TGF-β1 increased the expression levels of TRAP in addition to the expression of the major protease, CTSK (Figure 2). Table 1 shows the dose-response to the secretome, suggesting that 1 × 10^5^ cells failed to push osteoclastogenesis, whereas 1 × 10^7^ irradiated PBMCs/mL are also not supporting the process. Thus, the increase of osteoclastogenesis by the secretome corresponding to 1 × 10^6^ irradiated PBMCs/mL; irradiated PBMCs were verified at the level of gene expression.

Murine bone marrow-derived cells were exposed to various dilutions of the secretome from 1 × 10^5^ to 1 × 10^7^ irradiated PBMCs/mL in the presence of 30 ng/mL RANKL and 20 ng/mL M-CSF for five days. Gene expression analysis indicated the expression levels of TRAP and CTSK with the relative increase (x-fold) compared to M-CSF treated cells. Please note that it was a biphasic dose-response with only the secretome from 1 × 10^6^ irradiated PBMCs/mL (Sec10^6^) that enhanced osteoclastogenesis. Data from two independent experiments are shown (Exp1, Exp2). 

### 2.3. Secretome Contained TGF-β Activity based on a Bioassay with Primary Fibroblasts 

**Figure 3:**
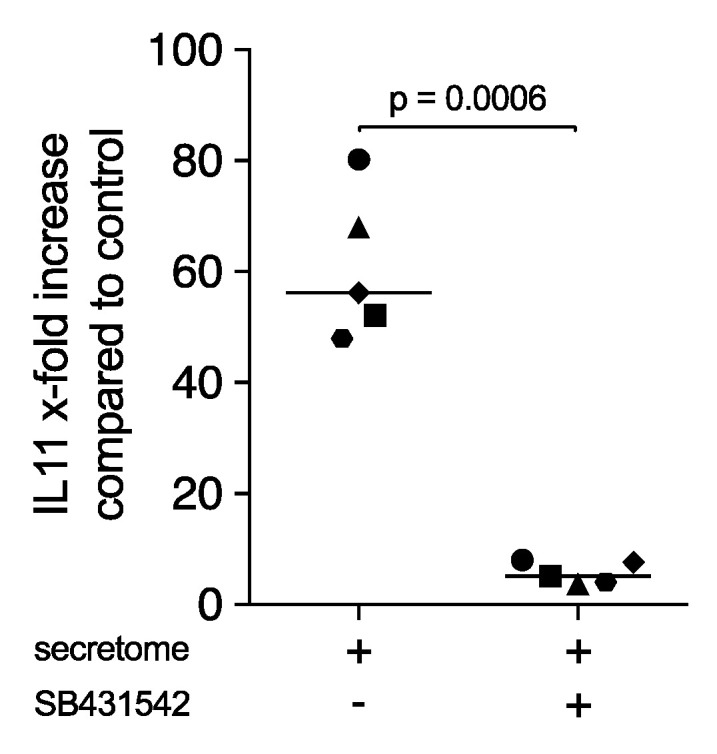
Secretome of irradiated peripheral blood mononuclear cells (PBMCs) increased IL11 expression in primary fibroblasts. Gingival fibroblasts were exposed to the secretome corresponding to 1 × 10^7^ irradiated PBMCs/mL in the presence and absence of 10 µm SB431542 for 24 h. X-fold change of IL11 expression compared to control was indicated. Dot plots represented independent experiments (*n* = 5). *p*-values were calculated by paired *t*-test.

To understand if the effects of the secretome can be mediated by TGF-β, we performed our established bioassay based on the TGF-β receptor I kinase-dependent increased expression of IL11 [34,35]. In support of the immunoassay findings [27,31], we showed that exposure of gingival fibroblasts to the secretome corresponding to 1 × 10^7^ PBMCs/mL increased the expression of IL11 in primary gingival fibroblasts (Figure 3). This concentration was 10 times higher than the one used for the macrophage culture but was ideal for inducing IL11 in fibroblasts. The TGF-β receptor I kinase antagonist SB431542 significantly decreased IL11 expression, which supported the presence of TGF-β activity in the secretome. Moreover, we performed an immunoassay that showed, on average, 120 pg/mL (min 111; max 190) TGF-β1 was found in the respective secretome. 

### 2.4. TGF-β Neutralizing Antibody Reduced the Secretome-Increased Osteoclastogenesis

**Figure 4:**
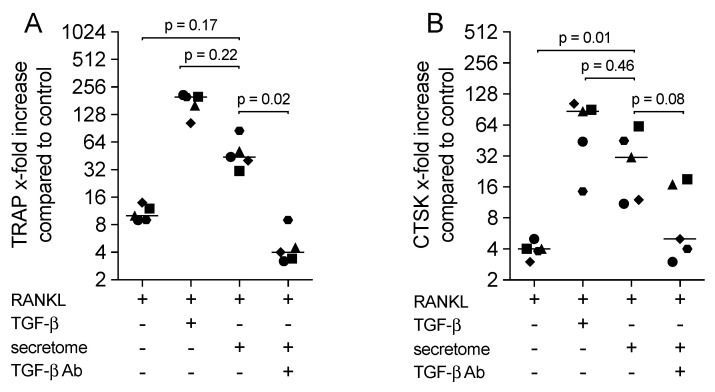
TGF-β neutralizing antibody reduced the secretome-increased osteoclastogenesis. Murine bone marrow-derived macrophages were exposed to the secretome corresponding to 1 × 10^6^ irradiated PBMCs/mL or 10 ng/mL TGF-β1 in the presence of 30 ng/mL RANKL and 20 ng/mL M-CSF, and a TGF-β neutralizing antibody (TGF-β Ab; 20 µg/mL) for five days. X-fold change of expression of TRAP (**A**) and CTSK (**B**) compared to M-CSF control was indicated. Dot plots represented independent experiments (*n* = 5). *p*-values were calculated by the Friedman test.

To investigate the possible involvement of secretome-derived TGF-β on osteoclastogenesis, TGF-β neutralizing antibodies were introduced. We showed that the presence of the TGF-β neutralizing antibody reduced the osteoclastogenic activity of the secretome indicated by the expression of TRAP and CTSK (Figure 4). Thus, the data supported a possible supportive role of the secretome-derived TGF-β on osteoclastogenesis in vitro. 

## 3. Discussion

Controlling the differentiation switch of hematopoietic progenitor towards the various cell lineages specialized to resolve inflammation and to resorb mineralized tissues is a fundamental principle of tissue homeostasis, regeneration, and repair. This controlling occurs at the local level by paracrine signals. It is the dying cells being predestined to provide a paracrine environment that favors the removal of the damaged tissue while supporting its replacement by new tissue. The secretome of irradiated PBMCs that underwent apoptosis and necroptotic cell death [18] can provide such a paracrine environment. This secretome has already proven to control the differentiation switch of hematopoietic progenitors to suppress dendritic cells formation [28] and to shift macrophages from an inflammatory M1 towards a resolving M2 phenotype [29]. In bone, it requires osteoclasts to remove damaged tissue before osteoblasts can rebuild the mineralized matrix [1]. The present research provided another piece of knowledge on how the secretome of irradiated PBMCs affects the differentiation switch of hematopoietic progenitors, in the present study, towards osteoclastogenesis in murine bone marrow cultures.

Our findings may have been predicted, knowing that recombinant TGF-β greatly pushes in vitro osteoclastogenesis [10] and that TGF-β in the ng/mL range was identified in the secretome of irradiated PBMCs [27,31]. We have extended this knowledge by showing the TGF-β activity based on the increased expression of IL11 in gingival fibroblasts that was greatly dependent on the activation of the TGF-β receptor I kinase. Detecting TGF-β in the secretome, however, did not predict its osteoclastogenic activity. Nevertheless, even though the secretome is a complex cocktail of protein and lipid mediators, our data suggested that it was indeed the secretome-derived TGF-β that pushed osteoclastogenesis in vitro as the presence of the neutralizing antibody almost completely blocked osteoclastogenesis. Thus, we can conclude that TGF-β was required to mediate the in vitro osteoclastogenic activity of the secretome of PBMCs.

The clinical relevance of these in vitro findings remains unclear. We have to assume that dying cells in the inflamed periodontal tissues [14,15,16,17], including those of the macrophage population, are possibly involved in the catabolic changes that culminate in the resorption of the alveolar bone. Under this premise, showing that the secretome of dying PBMCs supports osteoclastogenesis makes sense. Our experimental setting may have simulated a paracrine environment that supported the ongoing catabolic events of bone loss. This interpretation is somehow nihilistic as it puts the secretome in the light of catabolism, but accumulating evidence greatly supports a positive view on the secretome with respect to myocarditis [19], chronic heart failure [20], spinal cord injury [21], stroke [22], and wound healing [23]. Thus, we have to appreciate the paradigm that dying blood-born mononuclear cells, maybe in concert with the dying osteocytes [6], signal the need for bone removal before the anabolic process of renewal is initiated. In support of this paradigm, the secretome is stimulating angiogenesis [31], and angiogenesis is critically involved in bone regeneration [36]. Taken together, our in vitro observation should be interpreted in the concert of the beneficial effects already observed with the secretome of PBMCs. 

There are other study limitations that should be considered. The actual concentration of the secretome-derived TGF-β in the bone marrow culture is around 10–20 pg/mL [27,31], which is the least significant concentration to enhance osteoclastogenesis [10]. This concentration represents 10^6^ irradiated PBMCs/mL. In the TGF-β bioassay, we used the secretome from 10^7^ irradiated PBMCs/mL, which clearly confirmed the respective TGF-β activity. However, once we increased the concentration of the secretome to 10^7^ irradiated PBMCs/mL, the formation of osteoclasts was negatively affected. This observation was rather unexpected and might be explained by inhibitors of osteoclastogenesis in the secretome that exceeded a certain threshold. We also have to state that neither the decrease of osteoclastogenesis observed with the TGF-β neutralizing antibody nor with the TGF-β receptor I kinase inhibitor SB431542 (data not shown) provided full evidence that the effect of the secretome was mediated via TGF-β. Thus, we cannot rule out that the autocrine production of TGF-β by the bone marrow cells paves the way for the secretome to push osteoclastogenesis, maybe ligands independent of TGF-β. To provide further details, future studies should take advantage of the conditional deletion of TGF-β in macrophages using an inflammatory osteolysis model with macrophage apoptosis and necrosis. Alternatively, conditional deletion of TGF-β receptor signaling in osteoclast progenitors would help to test this hypothesis. Recent observations that necrotic osteocytes release spliceosome-associated protein 130, a macrophage-inducible C-type lectin ligand pushing osteoclastogenesis [6], may provide another molecular mechanism linking the secretome of dying cells with osteoclastogenesis. 

## 4. Material and Methods

### 4.1. Secretome of PBMCs

The preparation of the secretome was previously described in great detail, up to the level of viral clearance [24] and good manufacturing practice [25]. In brief, heparinized blood samples for PBMC isolation were obtained from healthy volunteers at the Austrian Red Cross Blood Transfusion Service of Upper Austria, Linz, Austria. All donors provided informed written consent. Briefly, PBMCs were isolated from heparinized blood using density gradient centrifugation via Ficoll-Paque PLUS (GE Healthcare Bio-Sciences AB, Umea, Sweden). The buffy coat that contained the PBMCs was resuspended at 2.5  ×  10^7^ cells/mL in CellGro serum-free medium (CellGenix, Freiburg, Germany), irradiated with Cesium-137 for 17 min (60 Gy), and cultivated for 24 hours [31]. Supernatants were collected by centrifugation at 400g for nine minutes and passed through 0.2 µm filters. Methylene blue treatment for viral clearance was performed as described previously [24]. Secretomes were lyophilized and terminally sterilized by high-dose γ-irradiation (Gammatron 1500, UKEM 60Co irradiator). Reconstitution was performed by serum-free Minimum Essential Medium Eagle-Alpha Modification (αMEM) to receive a stock concentration equivalent to 2 × 10^7^ cells/mL that was further diluted to a working concentration equivalent to or below 1 × 10^7^ cells/mL. All experiments were performed with batches A000918399096 and A000918399129, representing the secretome of 12 pooled donors. The protein, lipid, and miRNA composition of the secretome were reported [31], and immunoassays revealed TGF-β levels between 2.25 and 4.75 ng/mL [25].

### 4.2. Bone Marrow-Derived Osteoclastogenesis

BALB/c mice at the ages of 6–8 weeks were purchased from Animal Research Laboratories, Himberg, Austria. Euthanasia was performed with cervical dislocation immediately prior to the organ donation. Organ donation did not require formal ethical approval. The femora and tibiae of the mice were removed after scarifying, and bone marrow cells were collected. Bone marrow cells were seeded at 4 × 10^6^ cells/cm^2^ into 24-well plates and grown for five days in αMEM, supplemented with 10% fetal calf serum (FCS) and 1% antibiotics. Receptor activator of nuclear factor kappa-B ligand (RANKL, 30 ng/mL; ProSpec-Tany TechnoGene Ltd., Rehovot, Israel) and macrophage colony-stimulating factor (M-CSF, 20 ng/mL, ProSpec-Tany TechnoGene Ltd., Rehovot, Israel) were used to induce osteoclastogenesis. Control experiments [10] were performed in the presence of human transforming growth factor beta-1 (TGF-β1, 10 ng/mL; ProSpec-Tany TechnoGene Ltd., Rehovot, Israel) For test experiments, the secretome of irradiated PBMCs was included in the culture medium containing RANKL and M-CSF. In this setting, the TGF-β neutralizing pan-specific polyclonal rabbit IgG AB-100-NA (R&D Systems, Minneapolis, MN, USA) was used at 20 µg/mL and was used to block the TGF-β activity of the secretome. After five days, histochemical staining for tartrate-resistant acid phosphatase (TRAP) was performed following the instructions of the manufacturer (387A; Sigma-Aldrich, St. Louis, MO, USA). In brief, fixed cells were incubated in a solution of Naphthol AS-BI phosphoric acid and freshly diazotized Fast Garnet GBC. TRAP-positive cells stained red and were considered osteoclast when they had three or more nuclei. In parallel, total RNA was isolated.

### 4.3. TGF-β Bioassay and Immunoassay

To determine the TGF-β activity of the secretome, a functional bioassay with fibroblasts was performed [34,35]. Human gingival fibroblasts were prepared from explant cultures of three independent donors after approval of the Ethical Committee of the Medical University of Vienna (EK Nr. 631/2007). Cells were cultured in a humidified atmosphere at 37 °C in a growth medium that consisted of DMEM, 10% fetal calf serum, and 1% antibiotics (Invitrogen Corporation, Carlsbad, CA, USA). Cells were plated in a growth medium at 30,000 cells/cm^2^ into culture dishes. The following day, cells were exposed to the secretome corresponding to 1 × 10^7^ cells/mL or recombinant human TGF-β1 at 10 ng/mL in serum-free medium for 24 h, before IL11 expression analysis was performed. SB431542, a TGF-β receptor I kinase inhibitor, was used at 10 µM (Calbiochem, Merck Millipore, Billerica, MA, USA). The immunoassay human TGF-β1 DuoSet ELISA (DY240, R&D Systems, Inc., Minneapolis, MN, USA) was used to quantify TGF-β levels.

### 4.4. RT-PCR

Total RNA was isolated with the ExtractMe total RNA kit that included a DNase digestion step (Blirt S.A., Gdańsk, Poland). Reverse transcription was performed with the SensiFAST cDNA kit (Bioline, London, UK). Polymerase chain reaction was completed with the SensiFAST™ SYBR^®^ master mix (Bioline). Amplification was monitored on the CFX Connect™ Real-Time PCR Detection System (Bio-Rad Laboratories, CA, USA). Primer sequences are mTRAP_F 5′-TACCTGTGTGGACATGACC-3′, mTRAP_R 5′-CAGATCCATAGTGAAACCGC-3′; mCTSK_F 5′-TGTATAACGCCACGGCAAA-3′, mCTSK_R 5′-GGTTCACATTATCACGGTCACA-3′; mGAPDH_F 5′-AACTTTGGCATTGTGGAAGG-3′, mGAPDH_R 5′-GGATGCAGGGATGATGTTCT-3′; hGAPDH_F 5′-AAGCCACATCGCTCAGACAC-3′, hGAPDH_R 5′-GCCCAATACGACCAAATCC-3′. IL11 primer was from Bio-Rad (qHsaCEP0049951). The amplification protocol was an initial denaturation at 95 °C for 2 min, followed by 40 cycles of 95 °C for 5 s and 60 °C for 30 s. Primer was used at 0.5 µM or following the instruction of Bio-Rad for IL11. The mRNA levels were calculated by normalization to the housekeeping gene GAPDH using the ΔΔCt method and the CFX Maestro Software (Version 2.0, Bio-Rad Laboratories, CA, USA) for Real-Time PCR Systems. 

### 4.5. Statistical Analysis

All experiments were repeated at least three times. Data from individual experiments were shown as dot plots. Unless stated otherwise, data are described as x-fold change compared to unstimulated control. Statistical analysis was based on paired t-test and the Friedman test. Data were analyzed by the Prism 8.0E software (GraphPad Software; San Diego, CA, USA). Significance was set at *p* < 0.05.

## Figures and Tables

**Table 1:** Dose response of the secretome on the expression of osteoclast marker genes.

	RANKL	RANKL + Sec10^5^	RANKL + Sec10^6^	RANKL + Sec10^7^
TRAP (Exp1)	52	33	128	46
TRAP (Exp2)	8	7	67	11
CTSK (Exp1)	48	37	89	20
CTSK (Exp2)	36	31	62	36

## References

[1] Teitelbaum S.L. (2000). Bone resorption by osteoclasts. Science.

[2] Seeman E., Delmas P.D. (2006). Bone quality—The material and structural basis of bone strength and fragility. N. Engl. J. Med..

[3] Mbalaviele G., Novack D.V., Schett G., Teitelbaum S.L. (2017). Inflammatory osteolysis: A conspiracy against bone. J. Clin. Investig..

[4] Gruber R. (2019). Osteoimmunology: Inflammatory osteolysis and regeneration of the alveolar bone. J. Clin. Periodontol..

[5] Kennedy O.D., Laudier D.M., Majeska R.J., Sun H.B., Schaffler M.B. (2014). Osteocyte apoptosis is required for production of osteoclastogenic signals following bone fatigue in vivo. Bone.

[6] Andreev D., Liu M., Weidner D., Kachler K., Faas M., Gruneboom A., Schlotzer-Schrehardt U., Munoz L.E., Steffen U., Grotsch B. (2020). Osteocyte necrosis triggers osteoclast-mediated bone loss through macrophage-inducible C-type lectin. J. Clin. Investig..

[7] Farr J.N., Xu M., Weivoda M.M., Monroe D.G., Fraser D.G., Onken J.L., Negley B.A., Sfeir J.G., Ogrodnik M.B., Hachfeld C.M. (2017). Targeting cellular senescence prevents age-related bone loss in mice. Nat. Med..

[8] Acosta J.C., Banito A., Wuestefeld T., Georgilis A., Janich P., Morton J.P., Athineos D., Kang T.W., Lasitschka F., Andrulis M. (2013). A complex secretory program orchestrated by the inflammasome controls paracrine senescence. Nat. Cell Biol..

[9] Medina C.B., Mehrotra P., Arandjelovic S., Perry J.S.A., Guo Y., Morioka S., Barron B., Walk S.F., Ghesquiere B., Krupnick A.S. (2020). Metabolites released from apoptotic cells act as tissue messengers. Nature.

[10] Fuller K., Lean J.M., Bayley K.E., Wani M.R., Chambers T.J. (2000). A role for TGFbeta(1) in osteoclast differentiation and survival. J. Cell Sci..

[11] Herr B., Zhou J., Werno C., Menrad H., Namgaladze D., Weigert A., Dehne N., Brune B. (2009). The supernatant of apoptotic cells causes transcriptional activation of hypoxia-inducible factor-1alpha in macrophages via sphingosine-1-phosphate and transforming growth factor-beta. Blood.

[12] Chen W., Frank M.E., Jin W., Wahl S.M. (2001). TGF-beta released by apoptotic T cells contributes to an immunosuppressive milieu. Immunity.

[13] Vallion R., Bonnefoy F., Daoui A., Vieille L., Tiberghien P., Saas P., Perruche S. (2015). Transforming growth factor-beta released by apoptotic white blood cells during red blood cell storage promotes transfusion-induced alloimmunomodulation. Transfusion.

[14] Listyarifah D., Al-Samadi A., Salem A., Syaify A., Salo T., Tervahartiala T., Grenier D., Nordstrom D.C., Sorsa T., Ainola M. (2017). Infection and apoptosis associated with inflammation in periodontitis: An immunohistologic study. Oral Dis..

[15] Taskan M.M., Balci Yuce H., Karatas O., Gevrek F., Toker H. (2019). Evaluation of the effect of oleuropein on alveolar bone loss, inflammation, and apoptosis in experimental periodontitis. J. Periodontal Res..

[16] Curylofo-Zotti F.A., Elburki M.S., Oliveira P.A., Cerri P.S., Santos L.A., Lee H.M., Johnson F., Golub L.M., Rossa C.J., Guimaraes-Stabili M.R. (2018). Differential effects of natural Curcumin and chemically modified curcumin on inflammation and bone resorption in model of experimental periodontitis. Arch. Oral Biol..

[17] Qin X., Liu J.Y., Wang T., Pashley D.H., Al-Hashim A.H., Abdelsayed R., Yu J.C., Mozaffari M.S., Baban B. (2017). Role of indoleamine 2,3-dioxygenase in an inflammatory model of murine gingiva. J. Periodontal Res..

[18] Simader E., Beer L., Laggner M., Vorstandlechner V., Gugerell A., Erb M., Kalinina P., Copic D., Moser D., Spittler A. (2019). Tissue-regenerative potential of the secretome of gamma-irradiated peripheral blood mononuclear cells is mediated via TNFRSF1B-induced necroptosis. Cell Death Dis..

[19] Hoetzenecker K., Zimmermann M., Hoetzenecker W., Schweiger T., Kollmann D., Mildner M., Hegedus B., Mitterbauer A., Hacker S., Birner P. (2015). Mononuclear cell secretome protects from experimental autoimmune myocarditis. Eur. Heart J..

[20] Lichtenauer M., Mildner M., Hoetzenecker K., Zimmermann M., Podesser B.K., Sipos W., Berenyi E., Dworschak M., Tschachler E., Gyongyosi M. (2011). Secretome of apoptotic peripheral blood cells (APOSEC) confers cytoprotection to cardiomyocytes and inhibits tissue remodelling after acute myocardial infarction: A preclinical study. Basic Res. Cardiol..

[21] Haider T., Hoftberger R., Ruger B., Mildner M., Blumer R., Mitterbauer A., Buchacher T., Sherif C., Altmann P., Redl H. (2015). The secretome of apoptotic human peripheral blood mononuclear cells attenuates secondary damage following spinal cord injury in rats. Exp. Neurol..

[22] Altmann P., Mildner M., Haider T., Traxler D., Beer L., Ristl R., Golabi B., Gabriel C., Leutmezer F., Ankersmit H.J. (2014). Secretomes of apoptotic mononuclear cells ameliorate neurological damage in rats with focal ischemia. F1000Research.

[23] Mildner M., Hacker S., Haider T., Gschwandtner M., Werba G., Barresi C., Zimmermann M., Golabi B., Tschachler E., Ankersmit H.J. (2013). Secretome of peripheral blood mononuclear cells enhances wound healing. PLoS ONE.

[24] Gugerell A., Sorgenfrey D., Laggner M., Raimann J., Peterbauer A., Bormann D., Suessner S., Gabriel C., Moser B., Ostler T. (2020). Viral safety of APOSEC. Blood Transfus..

[25] Laggner M., Gugerell A., Bachmann C., Hofbauer H., Vorstandlechner V., Seibold M., Gouya Lechner G., Peterbauer A., Madlener S., Demyanets S. (2020). Reproducibility of GMP-compliant production of therapeutic stressed peripheral blood mononuclear cell-derived secretomes, a novel class of biological medicinal products. Stem Cell Res. Ther..

[26] Wuschko S., Gugerell A., Chabicovsky M., Hofbauer H., Laggner M., Erb M., Ostler T., Peterbauer A., Suessner S., Demyanets S. (2019). Toxicological testing of allogeneic secretome derived from peripheral mononuclear cells (APOSEC): A novel cell-free therapeutic agent in skin disease. Sci. Rep..

[27] Simader E., Traxler D., Kasiri M.M., Hofbauer H., Wolzt M., Glogner C., Storka A., Mildner M., Gouya G., Geusau A. (2017). Safety and tolerability of topically administered autologous, apoptotic PBMC secretome (APOSEC) in dermal wounds: A randomized Phase 1 trial (MARSYAS I). Sci. Rep..

[28] Laggner M., Copic D., Nemec L., Vorstandlechner V., Gugerell A., Gruber F., Peterbauer A., Ankersmit H.J., Mildner M. (2020). Therapeutic potential of lipids obtained from gamma-irradiated PBMCs in dendritic cell-mediated skin inflammation. EBioMedicine.

[29] Panahipour L., Kochergina E., Laggner M., Zimmermann M., Mildner M., Ankersmit H.J., Gruber R. (2020). Role for Lipids Secreted by Irradiated Peripheral Blood Mononuclear Cells in Inflammatory Resolution in Vitro. Int. J. Mol. Sci..

[30] Madel M.B., Ibanez L., Wakkach A., de Vries T.J., Teti A., Apparailly F., Blin-Wakkach C. (2019). Immune Function and Diversity of Osteoclasts in Normal and Pathological Conditions. Front. Immunol..

[31] Wagner T., Traxler D., Simader E., Beer L., Narzt M.S., Gruber F., Madlener S., Laggner M., Erb M., Vorstandlechner V. (2018). Different pro-angiogenic potential of gamma-irradiated PBMC-derived secretome and its subfractions. Sci. Rep..

[32] Yasuda H., Shima N., Nakagawa N., Yamaguchi K., Kinosaki M., Mochizuki S., Tomoyasu A., Yano K., Goto M., Murakami A. (1998). Osteoclast differentiation factor is a ligand for osteoprotegerin/osteoclastogenesis-inhibitory factor and is identical to TRANCE/RANKL. Proc. Natl. Acad. Sci. USA.

[33] Drake M.T., Clarke B.L., Oursler M.J., Khosla S. (2017). Cathepsin K Inhibitors for Osteoporosis: Biology, Potential Clinical Utility, and Lessons Learned. Endocr. Rev..

[34] Panahipour L., Tabatabaei A.A., Gruber R. (2020). Hypoallergenic infant formula lacks transforming growth factor beta activity and has a lower anti-inflammatory activity than regular infant formula. J. Dairy Sci..

[35] Panahipour L., Stahli A., Haiden N., Gruber R. (2018). TGF-beta activity in cow milk and fermented milk products: An in vitro bioassay with oral fibroblasts. Arch. Oral Biol..

[36] Stegen S., Carmeliet G. (2018). The skeletal vascular system—Breathing life into bone tissue. Bone.

